# Moderate Physical Exercise Activates ATR_2_ Receptors, Improving Inflammation and Oxidative Stress in the Duodenum of 2K1C Hypertensive Rats

**DOI:** 10.3389/fphys.2021.734038

**Published:** 2021-10-14

**Authors:** Alda Cássia Alves da Silva, Juliana Soares Severo, Brenda Lois Barros dos Santos, Pedro Henrique Moraes Mendes, Lívia Maria Soares Nobre, Ana Patrícia de Oliveira, Francisco Cleber Silva Ferreira, Jand Venes Rolim Medeiros, Roberto Cesar Lima-Junior, Alexandre Havt, Raimundo Campos Palheta-Junior, Armênio Aguiar dos Santos, Moisés Tolentino

**Affiliations:** ^1^Graduate Program in Pharmacology, Federal University of Piauí, Teresina, Brazil; ^2^Laboratory of Exercise and Gastrointestinal Tract, Department of Physical Education, Federal University of Piauí, Teresina, Brazil; ^3^Graduate Program in Food and Nutrition, Federal University of Piauí, Teresina, Brazil; ^4^Department of Physiology and Pharmacology, School of Medicine, Federal University of Ceará, Fortaleza, Brazil; ^5^Graduate Program in Biotechnology, Federal University of Delta do Parnaíba, Parnaíba, Brazil; ^6^School of Veterinary Medicine, Federal University of Vale do São Francisco, Petrolina, Brazil

**Keywords:** angiotensin II, inflammation, oxidative stress, physical exercise, 2K-1C hypertension

## Abstract

**Background:** In addition to the cardiovascular and renal systems, the gastrointestinal tract also contains angiotensin ATR_1a_, ATR_1b_, and ATR_2_. We previously observed that the 2Kidney-1Clip hypertension model elicits physical exercise and gastrointestinal dysmotility, which is prevented by renin-angiotensin system blockers. Here, we investigate the effect of physical exercise on inflammation, stress biomarkers, and angiotensin II receptors in the duodenum of 2K1C rats.

**Methods:** Arterial hypertension was induced by the 2K1C surgical model. The rats were allocated in Sham, 2K1C, or 2K1C+Exercise groups. One week after surgery, they were submitted to a physical exercise protocol (running 5x/week, 60min/day). Next, we assessed their intestinal contractility, cytokine levels (TNF-α, IL-1β, and IL-6), oxidative stress levels (MPO, GSH, MDA, and SOD), and the gene expression of angiotensin receptors (ATR_1A_, ATR_1B_, and ATR_2_).

**Results:** In comparison with the Sham group, the 2K1C arterial hypertension decreased (*p*<0.05) the intestinal contractility. In comparison with 2K1C, the 2K1C+Exercise group exhibited lower (*p*<0.05) MPO activity (22.04±5.90 vs. 78.95±18.09 UMPO/mg tissue) and higher (*p*<0.05) GSH concentrations in intestinal tissues (67.63±7.85 vs. 31.85±5.90mg NPSH/mg tissue). The 2K1C+Exercise group showed lower (*p*<0.05) cytokine levels in the intestine than 2K1C rats. In comparison with the Sham group, the 2K1C+Exercise rats showed higher (*p*<0.05) gene expression of ATR_2_ in the duodenum.

**Conclusion:** 2K-1C hypertension elicits an oxidative stress and inflammation process in the duodenum. Physical exercise modulates the expression twice as much of ATR2 receptors, suggesting possible anti-inflammatory and antioxidant effects induced by exercise.

## Introduction

Arterial hypertension is a multifactorial clinical condition characterized by sustained elevation of systolic and diastolic blood pressure levels (Oparil et al., 2018). Its etiology may be due to vascular changes (Moraes and Tibirica, 2017), microglial activation in the central nervous system (Li and Wang, 2017), autonomic imbalance (Maki-Petaja et al., 2016), and dysfunction of renal mechanisms involved with blood pressure control, in particular the renin-angiotensin-aldosterone system (RAAS; Riet et al., 2015; Malha et al., 2018).

There are several experimental models used to shed light on the pathophysiology of arterial hypertension (Leong et al., 2015). Among them, the 2-kidney-1-clip (2K1C) model has shown notable similarity to renovascular hypertension in humans (Oboshi et al., 2016). The insertion of a silver clip in the kidney to constrict the renal artery causes supra-regulation of the RAAS, eliciting a chronic increase in the blood pressure values (Goldblatt et al., 1934).

According to a description of Oliveira-Sales et al. (2014), Gromotowicz-Poplawska et al. (2018), and Roncari et al. (2018), the hypertensive state is sustained within 5weeks, due to the increase in plasma concentrations of renin and, consequently, the increase in the production of angiotensin II and its action on AT1 receptors.

The RAAS is a well-known pathway of hormonal signaling related to the hydroelectrolytic balance and regulation of cardiovascular function that culminates in the formation of the octapeptide angiotensin II. Angiotensin II acts on G protein-coupled receptors, and when present in excess, it promotes an imbalance in the baroreflex system and increases cardiac contraction strength by influencing the electrical properties of myocytes and by causing changes in the sympathetic balance (Bruce and Kloet, 2017; Ferrario and Mullick, 2017).

Besides its well-known effects on the cardiovascular, renal, and nervous systems, the RAAS also modulates the gastrointestinal tract physiology, altering the intestinal permeability to salt and water as well as the gut motor behavior (Sharma et al., 2019; Tanaka and Itoh, 2019).

In general, the angiotensin receptors are characterized in types 1 and 2. In mammals, there are AT1 and AT2 angiotensin receptors, even though it is not yet possible to differentiate the subtypes (AT1a and AT1b). On the other hand, rats already express two AT1a and AT1b subtypes, with AT1a being mainly expressed in the lung, vascular smooth muscle, and hypothalamus, and the AT1b type expressed in the adrenal and specific regions of the brain (Zhu et al., 1998; Premer et al., 2013).

By means of its type 1A (ATR_1A_), type 1B (ATR_1B_), and type 2 (ATR_2_), angiotensin II has a direct effect on intestinal smooth muscle cells (Fandriks, 2011; Garg et al., 2012). Angiotensin II can also impact the gastrointestinal tract motility by increasing the sympathetic input, with repercussions on the enteric nervous system. Thus, some pharmacological and non-pharmacological therapies have been proposed to mitigate the effects of angiotensin II on gastrointestinal dysmotility (Mastropaolo et al., 2015; Zizzo et al., 2016; Li and Wang, 2017; He et al., 2019).

We previously showed that 2K1C hypertension increases the gastric retention of a liquid test meal in awake rats, prevented by treatment with aliskiren, captopril or losartan, blockers of renin, angiotensin-converting enzyme (ACE), and ATR_1_, respectively (Lima et al., 2018). We also found that the performance of chronic physical exercise also reverted the 2K1C hypertension-induced gastric emptying delay, in the same way as treatment with these drugs. Therefore, physical exercise may be considered a non-pharmacological therapy for the control of gastrointestinal dysmotility associated with angiotensin II overactivity, due to its ability to reduce the activation of RAAS and balance the autonomic nervous system, by reducing sympathetic tone and increasing vagal cholinergic function (Fu and Levine, 2013; Li and Wang, 2017; Silva et al., 2017).

In addition, renovascular hypertension can elicit inflammation and oxidative stress in gastrointestinal tract tissues, manifested by increased secretion of proinflammatory cytokines and reactive oxidative species, which are attributed to increased secretion of corticoids, altered balance of microbiota and Angiotensin II receptor signaling, particularly AT1 activation in smooth muscle cells located in the gut, contributing to gastrointestinal dysfunction (Montezano et al., 2014; Li et al., 2016; Sharma et al., 2019; Tanaka and Itoh, 2019).

However, there is scarce evidence in the literature about the functional relationship between moderate physical exercise, RAAS, and gastrointestinal motility in the context of 2K1C hypertension. In this sense, we hypothesized that 2K1C hypertension would induce intestinal inflammation and an oxidative stress process and that the physical exercise could improve intestinal inflammation and oxidative stress *via* overexpression of the angiotensin receptors.

## Materials and Methods

### Animals and Ethical Approval

**Figure 1:**
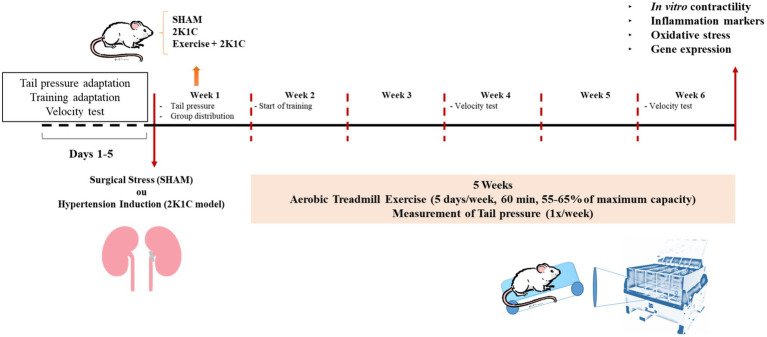
Experimental design.

Male Wistar rats weighing between 180 and 220g (*n*=5–9) were supplied by Federal University of Piauí, Brazil. All experiments were conducted according to “3R” principles. The animals were housed in collective cages with free access to water and food, with controlled temperature (25±2°C) and 12/12h light/dark cycle. All procedures were performed in accordance with the recommendations of the “Guide for the Care and Use of Laboratory Animals” (Clark et al., 1997) after approval by the Ethics Committee on Animal Use (CEUA) of Federal University of Piauí (Protocol 518/18). Rats were randomly allocated in Sham, 2K1C, and 2K1C+Exercise groups. Figure 1 shows the experimental design of this study.

### Induction of 2K1C Renovascular Hypertension

Renovascular hypertension (2K1C) was originally designed in dogs by Goldblatt and co-workers (1934), and was later adapted for rats (Lima et al., 2018). After anesthesia with ketamine (80mg/kg i.m) and xylazine (20mg/kg, i.m.), we performed a midline laparotomy to access the left renal artery, which was partially occluded by a U-shaped silver clip (0.20mm ID) to induce renovascular hypertension (2K1C group). In the normotensive control group (Sham), the rats were subjected to the same surgical procedures except the left renal artery occlusion. Next, all rats were treated with ampicillin sodium (200mg/kg, i.m). Rats of the 2K1C groups with systolic blood pressure (SBP) values <140mmHg were discarded. In this study, the percentage of 2K1C rats that did not develop hypertension was 10%.

### Treadmill Protocol

The treadmill exercise followed the protocol described by Sharma et al. (2010). Initially, all rats underwent a period of adaptation to the treadmill, which consisted in running during 5days for 10min at 13m/min between 15:00 and 17:00h. Next, they were subjected to a maximum capacity running test for determination of the maximal velocity (m/min), maximal distance traveled (m), and exhaustion time (s). Each animal was placed in one of the treadmill compartments and subjected to running at 5m/min, a velocity that increased at 3min intervals until the rat presented fatigue signals and the running was stopped. Then, we calculated the mean velocity of the group, the parameter used to determine the target zone of training. Exercise was performed at 55–65% maximum exercise capacity, during 60min for 5weeks, with 2days of rest every week. At week 2 and week 6, a new velocity test was performed.

### Assessment of SBP

The SBP was assessed by the non-invasive method of tail plethysmography (Kumral et al., 2016). Initially, the rats underwent an adaptation period of 2days in a cylindrical container, to minimize eventual bias in recordings due to stress. This was performed once a day during the week before and 3days after surgery. Initially, the rats were heated during 10min in a wooden box containing a 150-watt lamp to promote caudal artery dilation. Next, they were placed in the containment cylinder. An occluder and a sensor were fitted to the proximal portion of each rat’s tail. At the time of testing, they were coupled to a PE-399 electric sphygmomanometer connected to a signal transduction system (Powerlab 4/20, AD Instruments) and to a computer containing suitable software (LabChart Pro 8.0, AD Instruments) for SBP continuous recording. The SBP was equal to the arithmetic mean of three consecutive measurements, performed every 2min. Baseline SBP levels were obtained 1week before surgery.

### Assessment of Carbachol (CCh) Responsiveness in Isolated Strips of Duodenum

After 5weeks, rats from the Sham, 2K1C, and 2K1C+Exercise groups were used for the *in vitro* experiments. Initially, they were killed by lethal overdose of anesthetic (thiopental 100mg/kg, i.p.). Next, they underwent midline laparotomy to remove the duodenum located at (~5cm) of the pyloric sphincter region. Isolated strips of duodenum (~2cm) were then mounted in chambers for isolated smooth muscle preparations and maintained in Tyrode’s solution at 37°C, pH 7.4 and constantly oxygenated with a carbogenic mixture (95% O_2_ and 5% CO_2_), according to the method described by Magalhães et al. (1998). The baseline tension applied to the tissues was 3g. The intestinal strips were fixed on stainless steel terminals and attached to a voltage transducer coupled to a signal amplifier (AECAD 1604, AQCAD 2.3.6 software, AVS Projetos, SP) to record isometric stresses. Nutritional solutions were changed every 15min for 1h to avoid interference from metabolites (MacDonnell et al., 2005). At the end of the stabilization period, the carbachol response protocol (CCh) was started by cumulative addition of 1nM, 10nM, 100nM, 1μM, 10μM, and 100μM CCh solutions to the organ bath chambers. The interval between carbachol concentrations was ~5min.

### Myeloperoxidase Analysis

Briefly, the duodenum tissues were homogenized in potassium buffer with 0.5% hexyltrimethylammonium bromide (1ml/100mg of tissue). Then, the homogenate was centrifuged at 4,500rpm for 20min. Myeloperoxidase (MPO) activity in the resuspended pellet was evaluated by measuring the change in absorbance at 450nm using o-dianisidine dihydrochloride and 1% hydrogen peroxide. The results were expressed as units of MPO per mg of tissue (UMPO/mg of tissue; Bradley et al., 1982).

### Reduced Glutathione (GSH) Analysis

The concentration of GSH in the duodenum tissue samples was analyzed according to the method described by Sedlak and Lindsay, 1968. To determine the levels of the non-protein sulfhydryl groups, samples between 50 and 100mg of the animals’ duodenum were homogenized at a concentration of 1ml of 0.02M ED2 for each 100mg of tissue. Aliquots of 400μl of the homogenate were mixed in 320μl of distilled water and 80μl of 50% trichloroacetic acid for protein precipitation to occur. Tubes containing the material were centrifuged for 15min at 3,000rpm/4°C. Then, 400μl of the supernatant was added to 800μl of 0.4M Tris buffer (pH 8.9) and 20μl of dithio-nitrobenzoic acid or Ellman’s reagent. Next, the mixture was stirred for 3min and its absorbance was read by a spectrophotometer at 412nm. The concentrations of the non-protein sulfhydryl groups were expressed in mg NPSH/mg tissue.

### Malondialdehyde Evaluation

Duodenum samples were homogenized in cold solution of 1.15% KCl (1ml/100mg of tissue). Briefly, 250μl of each homogenate was added to 1% phosphoric acid (H3PO4) and 0.6% thiobarbituric acid (aqueous solution). Then, this mixture was stirred and heated in a boiling water bath for 45min. Next, it was immediately cooled in an ice water bath followed by the addition of 4ml of n-butanol. This mixture was stirred, and the butanol layer was separated by centrifugation at 1,200rpm for 15min. Its optical density was determined both at 535 and 520nm, and the difference in optical density values was considered to be the value of thiobarbituric acid. The results are expressed in nanomoles per milligram of tissue (nmol/mg of tissue; Mihara and Uchiyama, 1978).

### Superoxide Dismutase Levels

Using duodenum samples, a 10% homogenate was prepared and centrifuged at 3,000rpm for 15min at 4°C. Subsequently, each sample was added to phosphate, L-methionine (20mM), Triton X-100 (1% v/v), hydroxylamine chloride (10mM), and EDTA (50μM) solution. The tubes were placed in a water bath at 37°C for5min. Riboflavin (50μM) was added, and all measurements were corrected in a white light box for 10min. The solution was then transferred to an ELISA plate, followed by the addition of the Griess reagent. This was performed in an ELISA reader at 550nm. For complete protein dosage, we used the Labtest kit. The value of the superoxide dismutase (SOD) unit (ug/ug of total proteins) was calculated (Das et al., 2000).

### Cytokine Measurements

Briefly, microtiter plates were coated overnight at 4°C with polyclonal anti-rat IL-1-β, IL-6, or TNF-α (4μg/ml, DuoSet ELISA Development kit, R&D Systems). After blocking the plates, the samples and standards were added at various dilutions in duplicate and incubated at 4°C for 24h. The plates were washed with buffer (0.01M phosphate, 0.05M NaCl, 0.1% Tween 20, pH 7.2). After washing, biotinylated sheep polyclonal anti-IL-1β, anti-IL-6, and/or anti-TNF-α (diluted 1:1000 with assay buffer containing 1% bovine serum albumin) was added to the wells. After further incubation at room temperature for 1h, the plates were washed and 50μl of a diluted solution (1:5000) of avidin-conjugated horseradish peroxidase was added to the wells. The color reagent o-phenylene-diamine (40 lg/well) was added 15min later, and the plates were incubated at 37°C in the dark for 15–20min. The enzyme reaction was stopped with H_2_SO_4_, and the absorbance was measured at 490nm (Cunha et al., 1993). The values are expressed as pictograms of cytokines per milligrams (ρg/mg of duodenum tissue).

#### RT-PCR Analysis

Rats submitted or not to exercise were killed 10min postprandially, and the duodenum was harvested for total RNA extraction. For RNA isolation, we followed the protocol described for TRIzol^®^ Reagent (Thermo Fisher, MA, United States). The yield and quality of total RNA were determined spectrophotometrically (NanoDrop 2000^™^ – Thermo Fisher, MA, United States) using the wavelength of 260nm and the ratio of 260/280nm, respectively. One microgram of RNA, diluted to a final volume of 20μl, was reversely transcribed into cDNA using the GoScript^™^ Reverse Transcriptase kit (Promega, WI, United States) with the Veriti^™^ 96-well thermal cycler (Thermo Fisher, MA, United States).

We investigated the relative gene expression of the angiotensin receptors (ATR_1a_, ATR_1b_, and ATR_2_) and normalized the data using the expression of TATA box binding protein and ubiquitin C as housekeeping genes. Real-time PCR analysis was performed using the QuantStudio^™^ 5 Real-Time PCR System (Thermo Fisher, MA, United States) and the GoTaq^®^ qPCR Master Mix (Promega, WI, United States). The specific primer sequences (5′–3′) for ATR_1a_ were forward – TTCACCCTGCCTCAGGATCT and reverse – GGCTTTGCTTGGTTACTCC (product size 101bp; GeneBank code – NM_030985.4); for ATR_1b_ were forward – ACTCTTTCCTACCGCCCTTC and reverse – TTAGCCCAAATGGTCCTCTG (product size 141bp; GeneBank code – NM_031009.2); and for ATR_2_ were forward – CTTCCATGTTCTGACCTTCTT and reverse – CGGTTTCCAACGAAACAATAC (product size 159bp; GeneBank code – NM_012494.3, as published by He et al. (2019). The specific primer sequences (5′–3′) for ubiquitin C were forward – TCGTACCTTTCTCACCACAGTATCTAG and reverse – GAAAACTAAGACACCTCCCCATCA (product size 82bp; GeneBank code – NM_017314.1, as published by Al-Dasooqi et al. (2010); and for TATA box were forward – TAATCCCAAGCGGTTTGCTG and reverse – TTCTTCACTCTTGGCTCCTGTG (product size 111bp; GeneBank code – NM_001004198.1, as published by Martínez-Beamonte et al. (2011). Thermal cycling of all genes was preceded by an initial denaturation step at 95°C for 2min, followed by 45cycles comprising a denaturing step at 95°C for 15s, and an annealing step at 60°C for 1min. After each reaction, we also performed melting curve analysis to evaluate the specificity of the PCR amplification. Each PCR reaction well contained a final volume of 15μl and included 1.5μl of cDNA and gene-specific primers at 800nM. Negative samples were run with autoclaved Milli-Q water as the template. The threshold cycle (TC), defined as the fractional PCR cycle number at which the fluorescence reached 10 times the baseline standard deviation, was used to compare the expression of all tested genes. The mathematical model described by Livak and Schmittgen (2001) was applied to calculate the relative expression based on SYBR green staining.

### Statistical Analysis

The Shapiro–Wilk test was employed to assess data normality, and the results of each group (*n*=5 to 9 rats) were expressed as the mean±SEM. Then, for the purpose of comparison between the groups studied, one-way or two-way ANOVA was performed, followed by the Tukey test for variables with normal distribution, and one-way or two-way ANOVA followed by the Kruskal-Wallis test for data with nonparametric distribution. EC_50_ values and quantitative relative mRNA expression were reported as median, minimum and maximum, and interquartile range (*n*=8). Data were compared with the Sham group and analyzed by the Mann–Whitney test. The difference was considered significant if the *p* value was <0.05, adopting a 95% confidence interval.

## Results

**Figure 2:**
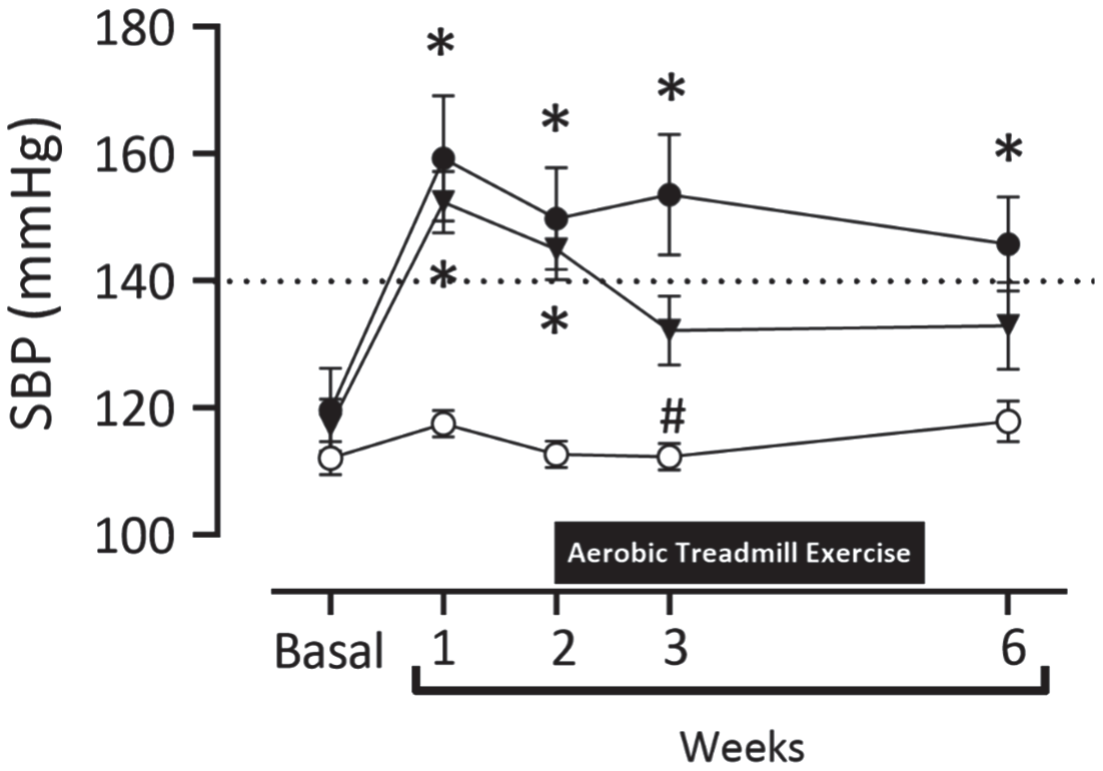
Systolic blood pressure values (mmHg) in normotensive and hypertensive rats by the 2K1C experimental model. Experimental groups: ○ Sham; ● 2K1C; and ▼ 2K1C+Exercise. Data are presented as mean ± SEM was used, two-way ANOVA, followed by Tukey’s test. Significance: *p*<0.05: (week 1, 2, 3, 6 –* vs. Sham); (week 3 – # vs. 2K1C).

In Figure 2 at baseline, there was no difference in systolic blood pressure between all groups (Sham: 112.1±2.6mmHg; 2K1C 119.7±6.5mmHg; Exercise+2K1C 117.3±4.1mmHg). Seven days after the induction of renovascular hypertension, there was a significant increase (*p*<0.05) in the SBP values of 2K1C and 2K1C+Exercise rats compared to the Sham group (2K1C 159.3±9.8mmHg and 2K1C+ Exercise 152.4±4.8mmHg vs. Sham 117.5±2.1mmHg). At week 3, there was a significant reduction (*p*<0.05) in the Exercise+2K1C group compared to the 2K1C group (Exercise +2K1C 132.2±5.5mmHg vs. 2K1C 153.6±9.4mmHg). At the end of week 6, there was a significant difference in the 2K1C group compared to Sham (145.8±7.3mmHg vs. 117.9±3.2mmHg). The Exercise+2K1C group does not show a significant difference from the Sham group in comparison with week 6.

**Figure 3:**
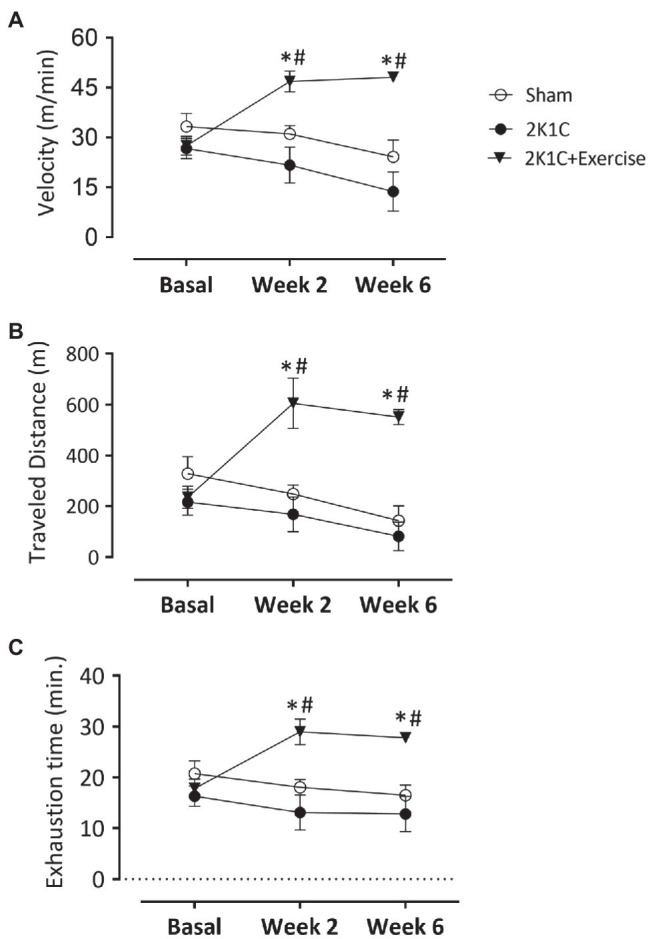
Monitoring parameters of treadmill training in normotensive and hypertensive 2K1C rats. Boxes: **(A)** velocity values (m/min); **(B)** distance covered (m); and **(C)** time of exhaustion (min) in normotensive or 2K1C hypertensive rats. Experimental groups: ○ Sham; ● 2K1C; and ▼ 2K1C+Exercise. Data are presented as mean±SEM was used, two-way ANOVA, followed by Tukey’s test. Significance: *p*<0.05: * vs. Sham; # vs. 2K1C.

Figure 3A shows the results of the maximal velocity test on the treadmill in all groups: Sham, 2K1C, and 2K1C+Exercise rats. In the basal situation, there was no difference between all groups in this test (Sham: 33.3±3.9, 2K1C: 26.6±3.0 and 2K1C+Exercise: 27.5±2.8m/min). In the second week, the 2K1C+Exercise rats showed a significant (*p*<0.05) increase of velocity compared to the Sham and 2K1C groups (Sham: 31.1±2.4m/min; 2K1C 21.6±5.4m/min; 2K1C+Exercise 46.8±3.1m/min). Similar increments were also obtained in the sixth week (Sham: 24.1±5.0m/min; 2K1C 13.7±5.9m/min; 2K1C+Exercise 48.1±1.6m/min; *p*<0.05).

Figure 3B shows the values of maximal distance traveled (m) on the treadmill in all groups. In the basal situation, there was no difference between all groups in this performance (Sham: 328.7±66.3, 2K1C: 216.8±51.0 and 2K1C+Exercise: 235.1±43.6m). In the second week, the 2K1C+Exercise rats showed a significant (*p*<0.05) improvement of maximal distance traveled (m) compared to the Sham and 2K1C groups (Sham: 248.7±35.4, 2K1C 168.5±68.0 and 2K1C+Exercise: 605.6±+98.54m). Similar increments were also obtained in the sixth week (Sham: 143.3±58.2, 2K1C: 81.5±56.9 and 2K1C+Exercise: 551.5±29.9m, p<0.05, respectively).

Figure 3C shows values of exhaustion time (min) on the treadmill in all groups. In the basal situation, there was no difference between all groups in this performance (Sham: 20.76±2.53m; 2K1C 16.32±1.99m; 2K1C+Exercise 17.83±1.87m). In the second week, the 2K1C+Exercise rats showed a significant (*p*<0.05) improvement of exhaustion time compared to the Sham and 2K1C groups (Sham: 18.06±1.51m; 2K1C 13.11±3.48m; 2K1C+Exercise 28.98±2.51s). Similar performances were also observed in the sixth week (Sham: 16.49±2.04m; 2K1C 12.81±3.81m; 2K1C+Exercise 27.81±0.87s; *p*<0.05).

**Figure 4:**
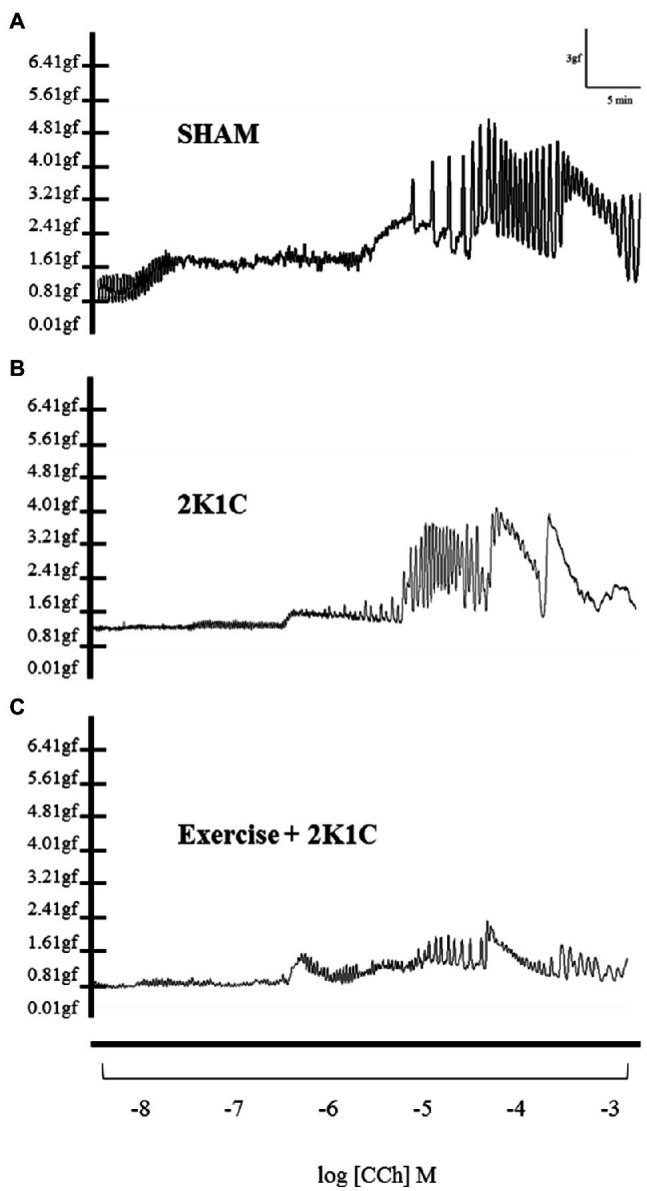
Original traces of contraction of segments of duodenum as concentrations of carbachol (-8M to -3M in log) in Sham **(A)**, 2K1C **(B)** and 2K1C+Exercise **(C)** rats.

Figures 4A–C show the typical recordings of all characteristics (traces) of the duodenum of the Sham, 2K1C, and 2K1C+Exercise rats in response to CCh stimulation (−8M to −3M in log). In comparison with the Sham rats, there was a substantial decrease in duodenal contractility in the 2K1C group and 2K1C+Exercise group.

**Figure 5:**
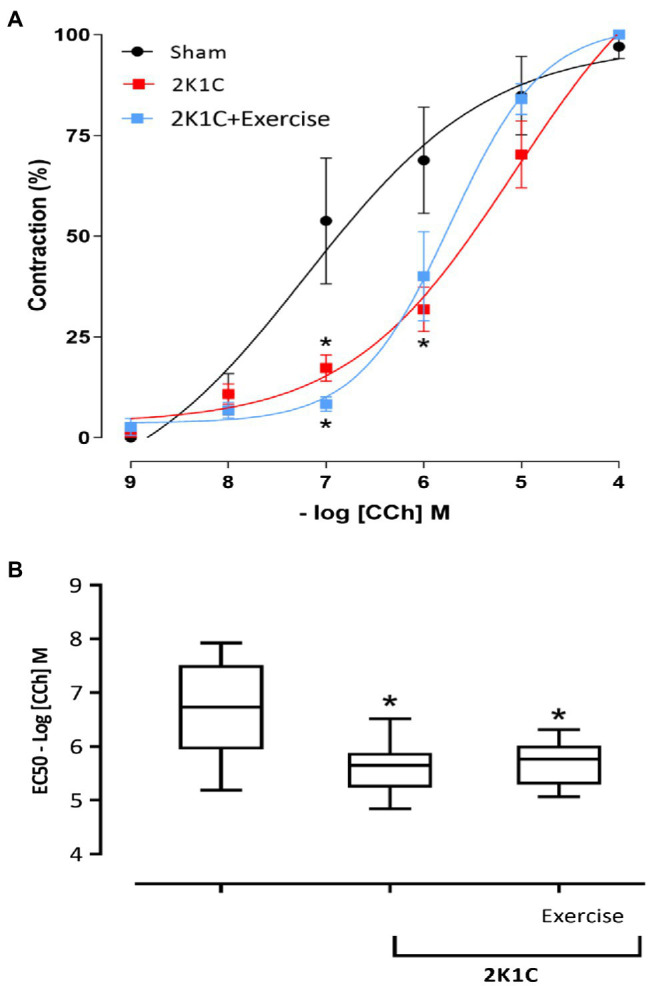
Responsiveness to Carbachol (CCh) in duodenum. **(A)** Cumulative curve of the effect of Carbachol (CCh) responsiveness in strips isolated from the duodenum (%); **(B)** median EC50 values [(25% percentile) – (75% percentile)] in strips isolated from the duodenum. Experimental groups: Sham, 2K1C, and 2K1C+Exercise. Results are described in mean±SEM; one-way or two-way ANOVA was used followed by Tukey’s test. Significance: *p*<0.05: * vs. Sham.

Figure 5A depicts the contractility of duodenal strips in response to CCh stimulation (CCh −9M to −4M in log). At 10^−7^ concentrations, the 2K1C and 2K1C+Exercise rats showed less responsiveness (*p*<0.05) to the cholinergic stimulus in comparison with the Sham group (2K1C 17.27±3.23%; 2K1C+Exercise 10.10±2.27% vs. Sham: 53.79±15.62%). At 10^−6^ concentration, there was also significantly (*p*<0.05) less responsiveness to the cholinergic stimulus of the 2K1C rats in comparison with the Sham group (2K1C 31.85±5.50 vs. Sham: 68.86±13.18%).

Figure 5B shows the comparison between the EC_50_ of all groups. Respective values are presented in median [(minimum) – (maximum)]. In comparison with the Sham group (− 6.73M) [(−5.18M) - (7.92M)], the CCh concentration that induced half of the maximum effect was significantly lower (*p*<0.05) in the 2K1C and 2K1C+Exercise groups, respectively (−5.65M) [(−4.84M) - (−6.51M)] and (−5.76M) [(−5.06M) – (−6.31M)].

**Figure 6:**
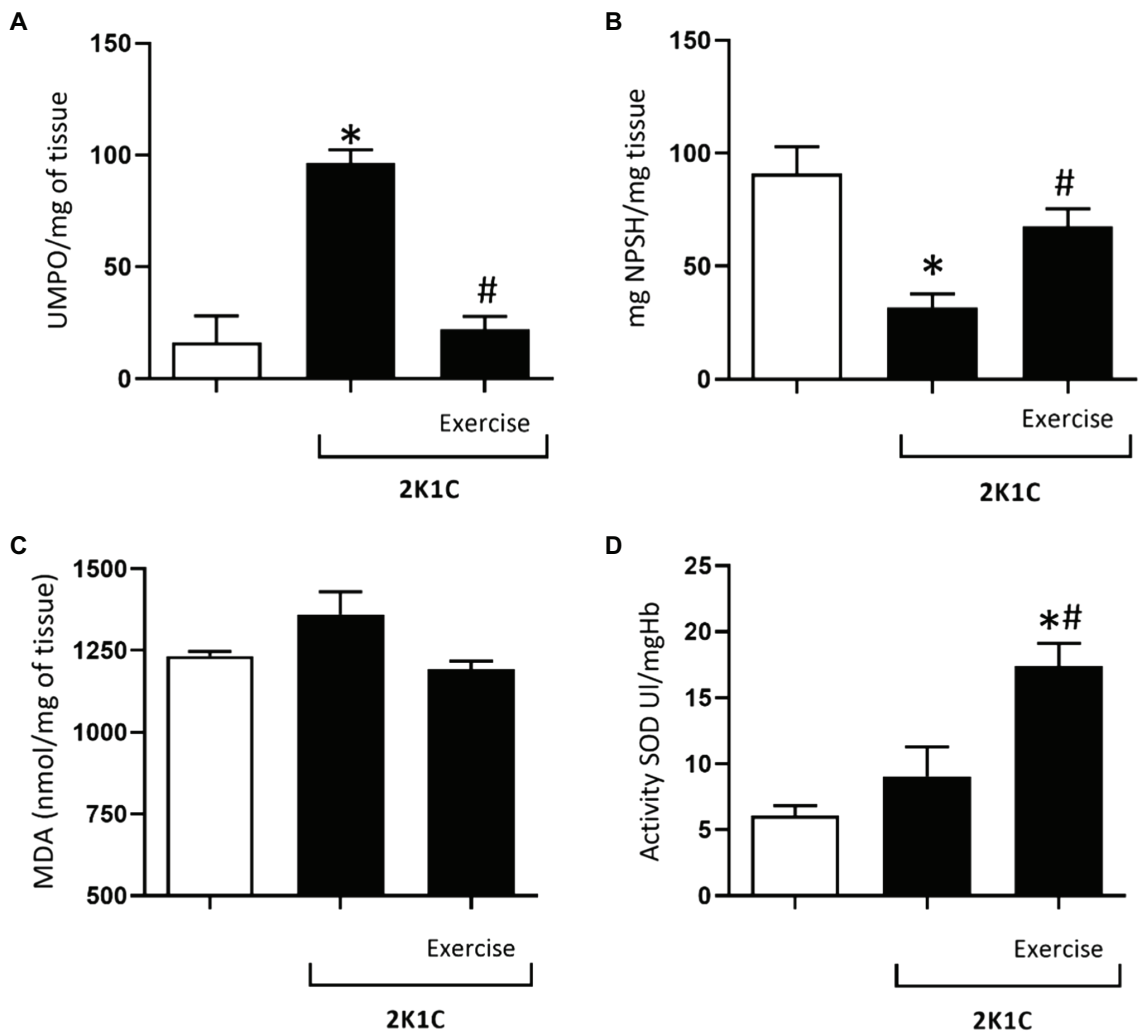
Myeloperoxidase (MPO) activity **(A)**, glutathione (GSH) concentrations **(B)**, malondialdehyde (MDA) concentrations **(C)**, superoxide dismutase (SOD) activity **(D)** in the duodenum of normotensive and hypertensive 2K1C rats. Experimental Groups: Sham; 2K1C; 2K1C + Exercise. For statistical analysis, mean ± SEM was used, by 2-way ANOVA, followed by Tukey or Kruskal-Wallis test. Significance: p <0.05: * vs. Sham, # vs. 2K1C.

Figure 6A shows the MPO activity in the duodenum of rats, with or without renovascular hypertension. In comparison with the Sham group, there was a significant increase (*p*<0.05) in the MPO activity in the duodenum of 2K1C rats (78.95±18.09 vs. 16.47±11.57 UMPO/mg tissue). On the other hand, this phenomenon was reverted in the 2K1C+Exercise group in comparison with the respective values of the 2K1C group (22.04±5.90 vs. 78.95±18.09 UMPO/mg tissue, *p*<0.05).

Figure 6B shows the glutathione (GSH) concentrations in the duodenum of the Sham, 2K1C, and 2K1C+exercise groups. In comparison with the Sham group, there was a significant decrease (*p*<0.05) in GSH concentration in the 2K1C group (31.8±5, 9 vs. 90.9±11.8mg NPSH/mg tissue). On the other hand, this phenomenon was reverted in the 2K1C+Exercise group compared with the respective values of the 2K1C group (67.63±7.8 vs. 31.85±5.90mg NPSH/mg tissue, *p*<0.05).

As can be seen in Figure 6C, there was no significant difference between the mean values of malondialdehyde (MDA) in the duodenum of rats with or without renovascular hypertension.

Figure 6D shows the activity of superoxide dismutase (SOD) in the duodenum of all groups. In comparison with the respective levels of the Sham group, there was a significant increase (*p*<0.05) in duodenal SOD values of the 2K1C+Exercise rats (14.5±2.1 Ul/mgHB vs. 6.08±0, 74 Ul/mgHB).

**Figure 7:**
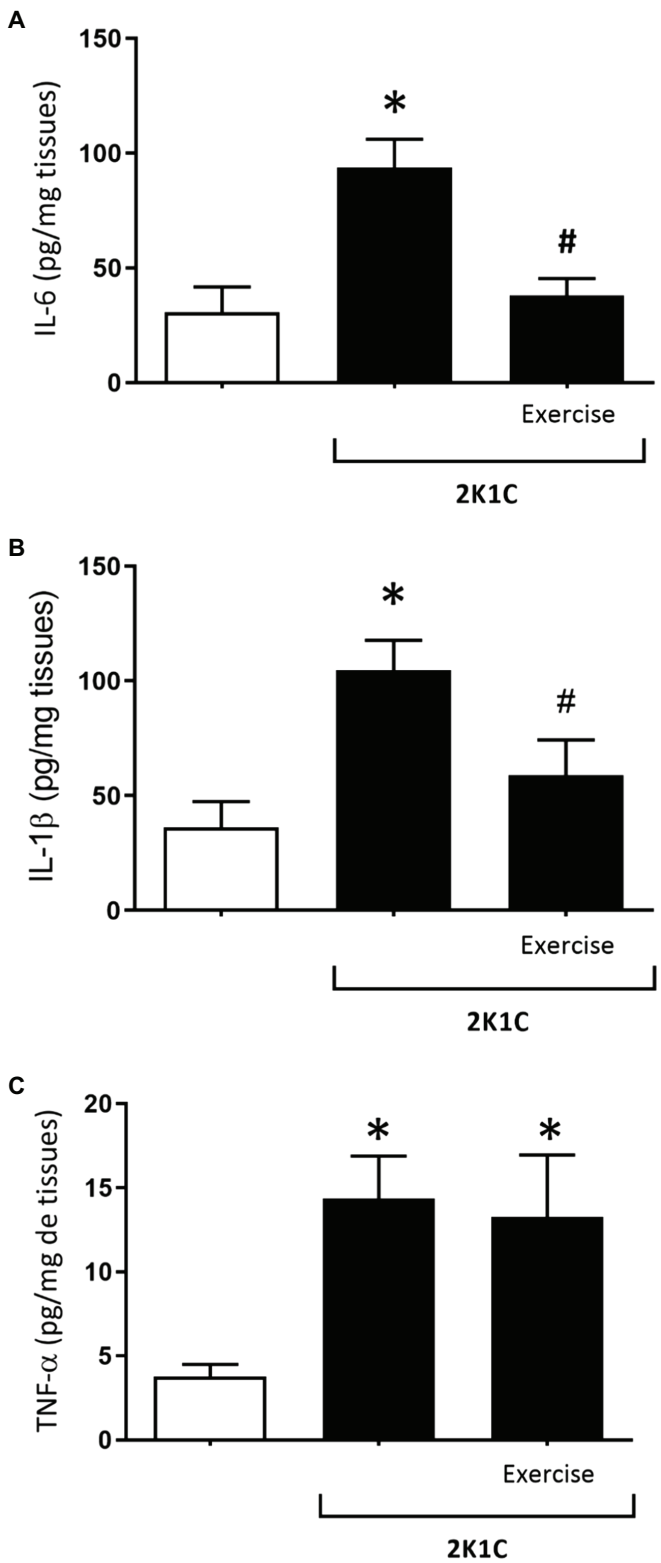
Concentrations of interleukin 6 (IL-6) **(A)**, interleukin (IL-1β) **(B)** and tumor necrosis factor alpha (TNF-α) **(C)** in the duodenum in hypertensive rats by the experimental model 2K1C. Experimental Groups: Sham; 2K1C; 2K1C + Exercise. For statistical analysis, mean ± SEM was used, by 2-way ANOVA, followed by Tukey or Kruskal-Wallis test. Significance: *p* <0.05: * vs. Sham, # vs. 2K1C.

Figure 7A shows the concentrations of IL-6 cytokine in the duodenum of rats, with or without renovascular hypertension. In comparison with the respective levels of the Sham group, there was a significant increase (*p*<0.05) in duodenal IL-6 values of the 2K1C rats (93.85±12.17 vs. 30.87±10.87 pg/mg tissue). On the other hand, the 2K1C+Exercise group showed a significant decrease (*p*<0.05) in duodenal IL-6 values compared to the 2K1C group (37.88±7.57 vs. 93.85±12.17 pg/mg tissue).

Figure 7B shows the concentrations of IL-1β cytokine in the duodenum of rats with or without renovascular hypertension. In comparison with the respective values of the Sham group, there was a significant increase (*p*<0.05) of the IL-1β values in the 2K1C rats (2K1C 104.7±13.03pg/mg tissue vs. Sham: 41.38±10.92pg/mg tissue). In turn, the 2K1C+ Exercise rats showed lower (*p*<0.05) IL-1 β values in comparison with the 2K1C group (2K1C+ Exercise 58.97±15.35pg/mg tissue vs. 2K1C 104.7±13.03pg/mg tissue).

Figure 7C shows the concentrations of TNF-α cytokine in the duodenum of rats with or without renovascular hypertension. In comparison with the respective values of the Sham group, there was a significant increase (*p*<0.05) of the TNF-α values in the duodenum of the 2K1C and 2K1C+Exercise rats (2K1C 14.36±2.53pg/mg tissue; 2K1C+Exercise 13.27±3.69pg/mg tissue vs. Sham 3.77±0.71pg/mg tissue).

**Figure 8:**
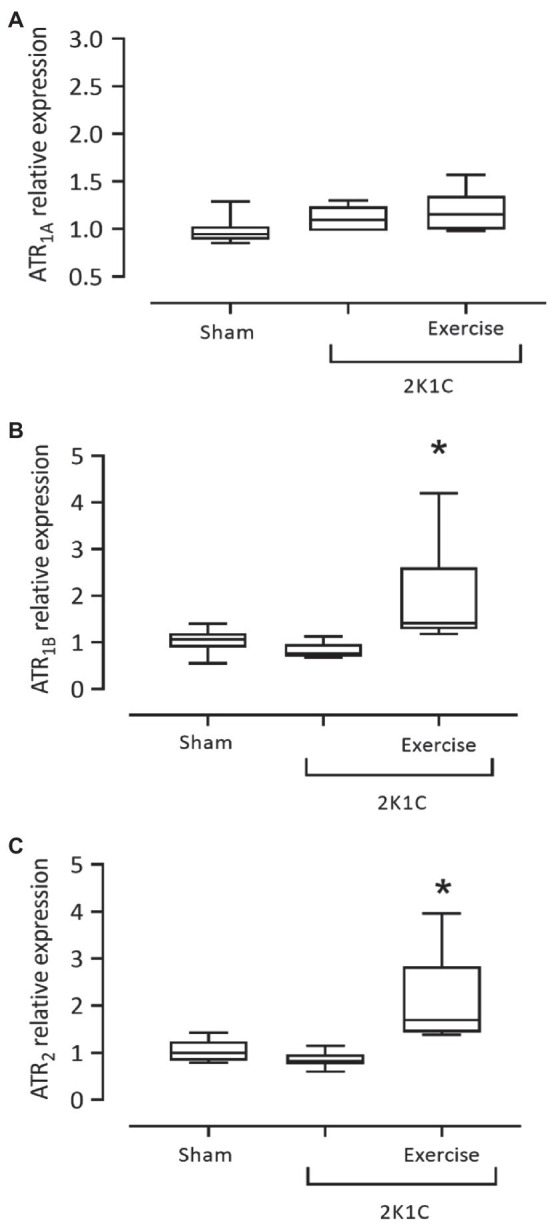
Gene expression of angiotensin II receptors, ATR1A **(A)**, ATR1B **(B)**, ATR2 **(C)** in the duodenum in hypertensive rats by the 2K1C experimental model. Experimental Groups: Sham; 2K1C; 2K1C + Exercise. For statistical analysis, mean ± SEM was used, by Mann-Whitney test. Significance: *p* <0.05: * vs. Sham.

Figure 8 shows the results of gene expression of ATR_1A_, ATR_1B_, and ATR_2_ receptors in the duodenum of rats with or without renovascular hypertension. There was no statistical difference between the mRNA expression for the ATR_1A_ receptor in all groups. However, the 2K1C+ Exercise rats exhibited higher (*p*<0.05) expression of the ATR_1B_ receptor in comparison with the respective values of the Sham group (2K1C+ Exercise: 1.415 [1.180–4.200] vs. Sham: 1.065 [0.550–1.40]). The 2K1C+ Exercise rats also exhibited higher (*p*<0.05) expression of the ATR_2_ receptor in comparison with the respective values of Sham group (2K1C+ Exercise: 1.690 [1.390–3.960] vs. Sham: 1,000 [0.790–1.430].

## Discussion

In the present study, we observed that moderate physical exercise improved the intestinal inflammation and oxidative stress, reducing IL-1β and IL-6 concentrations and MPO activity, and increasing GSH concentrations and SOD activity, as well as inducing overexpression of ATR_2_ angiotensin in the 2K1C hypertensive rats.

The two kidney one clip hypertension model, based on the constriction of the renal artery by inserting a silver clip (Goldblatt et al., 1934), was effective in raising the SBP values of rats in the present study. It was associated with increased concentration of angiotensin II, which acts on vascular AT1 receptors, maintaining the hypertensive state. In the present study, the physical exercise was able to mitigate the SBP increase from the second week of training. Similar results were observed by Waldman et al. (2017), where the voluntary dynamic exercise on steering wheels provided a considerable reduction in blood pressure in 2K1C rats.

Concerning the effect of the physical training, the rats submitted to exercise (2K1C+Exercise) showed notable improvement in speed, distance covered, and exhaustion time during the individual tests performed in weeks 2–6 (end of training), due to the body adaptations promoted by physical training, a well-known phenomenon described in the literature (Soares et al., 2011; Maia et al., 2015; Lima et al., 2020).

Carbachol (CCh) is a cholinergic agonist that binds to muscarinic receptors coupled to the G protein, specifically Gq/11, which releases calcium and consequently elicits smooth muscle contraction (Silva et al., 2014; Oliveira et al., 2017). Regarding the CCh responsiveness protocol of gastrointestinal tissues, there was a shift to the right of the contractility curve of the duodenum strips in 2K1C rats, indicating reduced responsiveness to the CCh cholinergic agonist. This phenomenon can be explained by the fact that renovascular hypertension increases the levels of angiotensin II, which can cause this hypo contractility (Zizzo et al., 2016).

It is well known that arterial hypertension is associated with activation of inflammatory pathways, exacerbating the generation of reactive oxygen species, leading to oxidative stress in the endothelium (Dinh et al., 2014). However, there are few studies of its impact on the gastrointestinal tract (Wunpathe et al., 2018; Qi et al., 2019). In the present study, we observed that renovascular hypertension increased the concentrations of IL-1β, IL-6, and TNF-α in the duodenum of 2K1C rats. We suggest that the increase in circulating angiotensin II, due to renovascular hypertension, is also associated with inflammation of the gastrointestinal tract, via activation of angiotensin ATR_1_ located in the duodenum circular and longitudinal layers, as well as in the myenteric plexus (Garg et al., 2012; He et al., 2019).

Interleukin-6 is a cytokine with dynamic regulation of health and disease. In hypertension, IL-6 can increase and act as a pro-inflammatory factor. On the other hand, this cytokine can also be secreted by the muscles after exercise-induced damage, stimulating muscle repair by concomitantly increasing IL-10, an anti-inflammatory cytokine (Petersen and Pedersen, 2005; Kumral et al., 2016). In the present study, physical exercise decreased the IL-6 concentrations in the duodenum of 2K1C+Exercise rats. Physical exercise’s effects on intestinal inflammation can also be mediated by an increase in the secretion of myokines with protective action, such as irisin, IL-10 and IL-1ra, and secretion of the glucagon-like peptide (GLP-1), which can repair the intestinal mucosa after damage due to physical stress (Bilski et al., 2016; Pedersen, 2017).

Commonly increased in arterial hypertension, interleukin-1β and TNF-α are cytokines associated with nuclear factor κB (NF-κB) translocation and transcription, which exacerbate inflammation, oxidative stress, and cell proliferation (Parpaleix et al., 2016; Verma et al., 2019). In our study, the 2K1C rats exhibited increased levels of IL-1β and TNF-α in the duodenum. These cytokines seem to be involved in the inflammasome complex in the gut, acting in conjunction with other pro-inflammatory cytokines, such as IL-6, which may contribute to increase the risk of intestinal malignancy (Mao et al., 2018). In our experimental conditions, physical exercise was able to reduce the IL-1β concentrations in the duodenum of 2K1C rats but not reduce the TNF-α in the duodenum. Thus, our results show that physical exercise has protective and anti-inflammatory properties, reducing the IL-6 and IL-1β concentrations in the duodenum of 2K1C hypertensive rats.

Regarding the oxidative stress markers, we observed an increase in MPO activity in the duodenum of 2K1C rats. MPO is an enzyme produced by the neutrophils in response to an inflammatory process (Bradley et al., 1982), and its gut activity can increase as a response to IL-8 signaling by tissue-resident macrophages, facilitating the recruitment of neutrophils to the gut (Chami et al., 2018; Kumar et al., 2018). We also found lower GSH concentrations in the duodenum of 2K1C rats when compared to control. GSH is a major component of endogenous antioxidant defense system in the gut (Barboza et al., 2017), a repercussion observed in the arterial hypertension, marked by altered balance of pro and antioxidant factors (Kumral et al., 2016).

No changes were observed in MDA concentrations and SOD activity in the duodenum of the 2K1C rats. Malondialdehyde is a product of lipid peroxidation and generally is increased in arterial hypertension (Verma et al., 2019), but no study has assessed this marker in the duodenum during hypertension. Regarding SOD, this enzyme forms the first line of defense against superoxide radicals, which form hydrogen peroxide (H_2_O_2_), and finally water (H_2_O) and oxygen (O_2_) by the subsequent enzymes (Santos et al., 2021). In arterial hypertension, angiotensin II can favor the generation of superoxide radicals through the activation of NADPH oxidase, but in the present study no effect was observed in the activity of SOD, an enzyme that contributes to neutralize this free radical (Li et al., 2016). All these facts notwithstanding, we observed that physical exercise prevents increased MPO activity in the duodenum of the 2K1C+Exercise rats. In addition, there was an increase in SOD activity in the 2K1C+Exercise group. Thus, one can infer that physical exercise protects the gastrointestinal tract mainly because of its actions to control oxidative stress (Qin et al., 2017), which can be noted by its role in reducing inflammation, as observed in the present study.

We also evaluated the expression of angiotensin receptors in the duodenum of 2K1C rats. The activation of ATR_1_ leads to NADPH oxidase activation, pro-inflammatory gene expression, cellular proliferation, vasoconstriction, and fibrosis (Montezano et al., 2014; Li et al., 2016). Angiotensin type 2 (ATR_2_) receptor activation stimulates endothelial oxide nitric synthase (eNOS), vasodilatation, antiproliferative, anti-inflammatory, and antioxidant action (Forrester et al., 2018). Spak et al. (2019) confirmed the presence of the RAA system in the duodenum, exercising functions in endothelial regulation, control of fluids and electrolyte balance, inflammation, and oxidative stress. In the present study, we did not observe hypertensive changes in the gene expression in ATR_1A_ or ATR_1B_ in the duodenum of 2K1C rats. However, physical exercise increased the expression of ATR_1B_ and ATR_2_ in the duodenum of these rats. We highlight the balance between these receptors’ expression, since running training promoted a substantial increase of ATR_2_ expression (about 2-fold greater than in 2K1C rats), which can explain the physical exercise’s effects on inflammation and oxidative stress attenuation observed in the present study, despite the increase of ATR_1B_. Shah et al. (2012) reported that swimming training increases the expression of ATR_2_ in the heart of 2K1C rats.

In this perspective, given the complexity of the mechanisms involved in the actions of physical exercise on renovascular hypertension-induced gastrointestinal tract dysmotility, it would be useful to investigate other biomarkers, such as the angiotensin (1–7) and ACE2, in the gastrointestinal tract, and the possible influence of mineralocorticoids, as well as to assess the nervous sympathetic activity in 2K1C rats, for a better understanding of such a relevant topic.

## Conclusion

**Figure 9:**
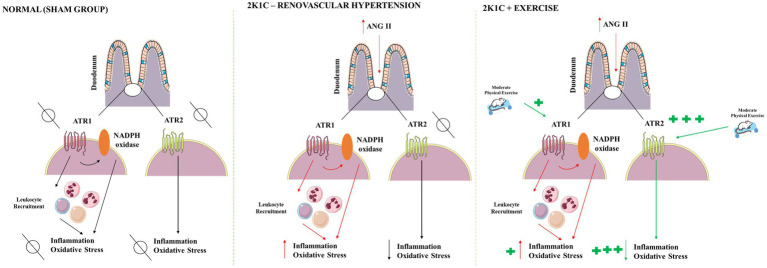
Proposed mechanism of physical exercise actions in renovascular hypertension. After activation of the renin-angiotensin-aldosterone system, the angiotensin II levels are raised in the blood. Angiotensin II acts on G protein-coupled receptors, mainly of the AT1 type; modulates functions in the GI tract; and activates NADPH oxidase which releases reactive oxygen species generating oxidative stress, in addition to promoting inflammation. Hypertension also decreases the CCh responsiveness of duodenum. Moderate physical exercise increases the expression of angiotensin receptors, particularly AT2 type, mediating anti-inflammatory, and antioxidant effects. In rats, 2K1C and submitted to physical exercise was not able to ameliorate the CCh responsiveness of duodenum. AT_1_, angiotensin II receptor type 1; AT_2_, angiotensin II receptor type 2; CCh, carbachol; NADPH, adenine and nicotinamide dinucleotide phosphate. Red arrows indicate hypertension effects. Green arrows indicate physical exercise effects.

There are many mechanisms that can explain the effects of exercise in the duodenum of hypertensive rats, which are illustrated in Figure 9. Renovascular hypertension showed reduced responsiveness to cholinergic stimulus with CCh in strips isolated from the duodenum. Exercise was not able to prevent the diminished response to CCh in 2K1C rats. Renovascular hypertension indicated an inflammatory process in the gastrointestinal tract, with increased levels of IL-1β and IL-6 and TNF-α in the duodenum. Physical exercise was able to significantly reduce the concentrations of the cytokines in this tissue. The presence of renovascular hypertension was also associated with oxidative stress markers, such as increased MPO activity and reduced GSH concentrations in the duodenum. Physical exercise reduced the oxidative stress markers, such as MPO activity, and caused an increase in GSH concentrations and antioxidant activity of SOD in the duodenum. In this study, physical exercise modulates angiotensin II ATR2 receptors in the duodenum of 2K1C rats suggesting the existence of anti-inflammatory and antioxidant effects mediated by this receptor.

## Data Availability Statement

The raw data supporting the conclusions of this article will be made available by the authors, without undue reservation.

## Ethics Statement

The animal study was reviewed and approved by Ethics Committee on Animal Use (CEUA) of the Federal University of Piauí (Protocol 518/18).

## Author Contributions

AS, JS, BS, and PM: all experimental protocols. JM, RL, and RP: provided some analysis and are majors’ contributors in writing the manuscript. AO, FF, and AH: gene expression analysis. AS and MT: supervision the paper, participated in the redaction and the review of the manuscript. All authors contributed to the article and approved the submitted version.

## Funding

This study was supported by grants from the Coordination for the Improvement of Higher Education Personnel (*Coordenação de Aperfeiçoamento de Pessoal de Nível Superior* *– CAPES*), CNPq (Edital Universal – CNPq. 421641/2018-5), and Piauí Research Support Foundation (*Fundação de Amparo à Pesquisa do Piauí – FAPEPI*).

## Conflict of Interest

The authors declare that the research was conducted in the absence of any commercial or financial relationships that could be construed as a potential conflict of interest.

## Publisher’s Note

All claims expressed in this article are solely those of the authors and do not necessarily represent those of their affiliated organizations, or those of the publisher, the editors and the reviewers. Any product that may be evaluated in this article, or claim that may be made by its manufacturer, is not guaranteed or endorsed by the publisher.
